# Wuli-Shili-Renli methodology as a service quality management tool among private older adults care institutions in Taizhou, China: an empirical study

**DOI:** 10.3389/fpubh.2026.1824122

**Published:** 2026-08-04

**Authors:** Lili Huang, Jing-Jing Fu, Dan Xu

**Affiliations:** 1 Department of Quality Control, Taizhou University Affiliated First People’s Hospital, Taizhou, Zhejiang, China; 2 Department of Nursing, Wenling Renxie Rehabilitation Hospital, Wenling, Zhejiang, China; 3 Department of Nursing, Taizhou First People’s Hospital, Taizhou, Zhejiang, China

**Keywords:** private older population care institutions, resource allocation, service capacity, service quality management, Wuli-Shili-Renli methodology

## Abstract

**Background:**

Population aging, a global crisis, has made private integrated medical - nursing facilities the primary providers for older adults care. However, the operational flaws of these facilities can potentially be addressed by the China - originated Wuli-Shili-Renli (WSR) methodology, which integrates physical resources, systematic management, and human factors.

**Methods:**

A retrospective empirical before-after study was conducted to evaluate the implementation effect of the WSR framework on service quality management in one representative private integrated medical-nursing care institution in Taizhou between 2021 and 2024. All outcome indicators adopted in this study were longitudinal annual monitoring data independently collected from this single institution. Pre- and post-implementation data were compared to assess improvements in service quality across different dimensions of the WSR framework.

**Results:**

Statistically significant improvements were associated with WSR framework implementation: in Service Resources (Wuli), the 24-h on-duty rate of clinical doctors increased from 71.51% to 100% (*p* < 0.001) and the clinical nurse–patient ratio improved from 15.14% to 21.58% (*p* < 0.001); in Service Operation (Shili), psychological/psychiatric support services were established (0 to 19 person-times, *p* < 0.001), social organization participation increased by 300% (3 to 12 annual visits, *p* = 0.035), and pipeline nursing staff nearly doubled (66 to 121, *p* < 0.001); in Service Evaluation (Renli), the older population admission rate rose from 76.5% to 80.5% (*p* < 0.001).

**Conclusion:**

The WSR-based model was associated with optimized resource allocation, standardized processes, and improved evaluation, providing a scalable path for the high-quality development of geriatric care services amid population aging.

## 1 Introduction

Population aging has emerged as a transnational demographic crisis reshaping global healthcare systems and social policies. According to the United Nations’ World Population Prospects 2024, the global population aged 60 and above will surge from 8.2 billion people in 2024 to around 10.3 billion in the mid-2080s, with the vast majority residing in low-income and middle-income countries (1). Restricted by rigid funding arrangements, standardized service patterns and limited capacity to meet diverse needs, public older adults care systems encounter notable operational challenges (2). Creating a gap that positions private integrated medical - nursing facilities as indispensable stakeholders (3). Benefiting from market-oriented operational flexibility, private older adults care institutions are capable of delivering customized services to meet diverse demands, including culturally inclusive care and technology-supported service modes (4, 5). They have pioneered community-integrated care and telemedicine service models, which have effectively reduced hospital readmission rates in high-income nations (2). Underscoring their irreplaceable role in advancing universal older adults care coverage. Additionally, they bridge major gaps in older adults care services across low- and middle-income countries by supplying a large share of long-term care services for older adults (6, 7).

Yet these indispensable providers worldwide are beset by systemic bottlenecks that hamper their ability to deliver consistent, high-quality care. China’s unique demographic trajectory not only amplifies these global challenges but also presents distinct contextual imperatives that demand targeted scholarly inquiry. As the world’s most populous aging society, China is home to 297 million people aged 60 and above, accounting for 21.1% of its total population (8). Approximately 50 million older adults people in China need integrated medical and nursing services due to chronic diseases or functional limitations, including about 35 million disabled older adults and 15 million dementia people over 65, with 78% of the older adults suffering from at least one chronic disease (9). Public older adults care institutions are constrained by limited operational capacity and can only meet a small fraction of this surging demand. This makes private integrated medical-nursing facilities the primary service providers in the domestic market (10). Nevertheless, China’s private integrated medical-nursing facilities are still plagued by prominent operational flaws, such as disjointed resource integration and poor coordination between clinical treatment and daily care service modules (10, 11).

To address this global research gap and contextual imperative, this study introduces the China-originated Wuli-Shili-Renli (WSR) systematic methodology for service quality management of private integrated medical-nursing facilities. Unlike one-dimensional tools such as the Balanced Scorecard (12) or lean healthcare (13), WSR integrates three core dimensions: Wuli (physical resources), Shili (systematic management), and Renli (human factors) (14). It extends health systems theory by integrating resource allocation, process standardization, and human-centered care into a unified analytical model, moving beyond fragmented approaches that treat these dimensions in isolation (15). By operationalizing WSR in this context, the study aims to provide a cross-culturally replicable framework for sustainable global older adults care.

## 2 Materials and methods

### 2.1 Study design

We adopt a retrospective empirical study approach to evaluate the implementation effect of the WSR methodology on service quality management among private integrated medical-nursing older adults care institutions in Taizhou from January 1, 2022 to December 31, 2023. Retrospective empirical methods are commonly employed to assess the effectiveness of management interventions by comparing pre- and post-implementation data (16). This approach enables the collection of objective metrics related to resource allocation and service capacity, thereby providing credible evidence for verifying the value of the WSR framework in practical settings (17).

### 2.2 Setting

This study was conducted in three private integrated medical and nursing care facilities in Taizhou from January 1, 2022 to December 31, 2023. All facilities are equipped with specialized medical and nursing teams, providing long-term care, chronic disease management, health check-up and other services.

### 2.3 Inclusion and exclusion criteria

Inclusion criteria: (1) The operation of the facility complies with the requirements of the integrated medical and nursing care model (18); (2) The facility has a stable management team; (3) The facility can provide complete medical and nursing data. Exclusion criteria: (1) The facility withdrew from the service system during the research period; (2) The facility has serious data record missing; (3) The facility experienced major safety accidents or management changes during the research period.

### 2.4 WSR development

**Figure 1:**
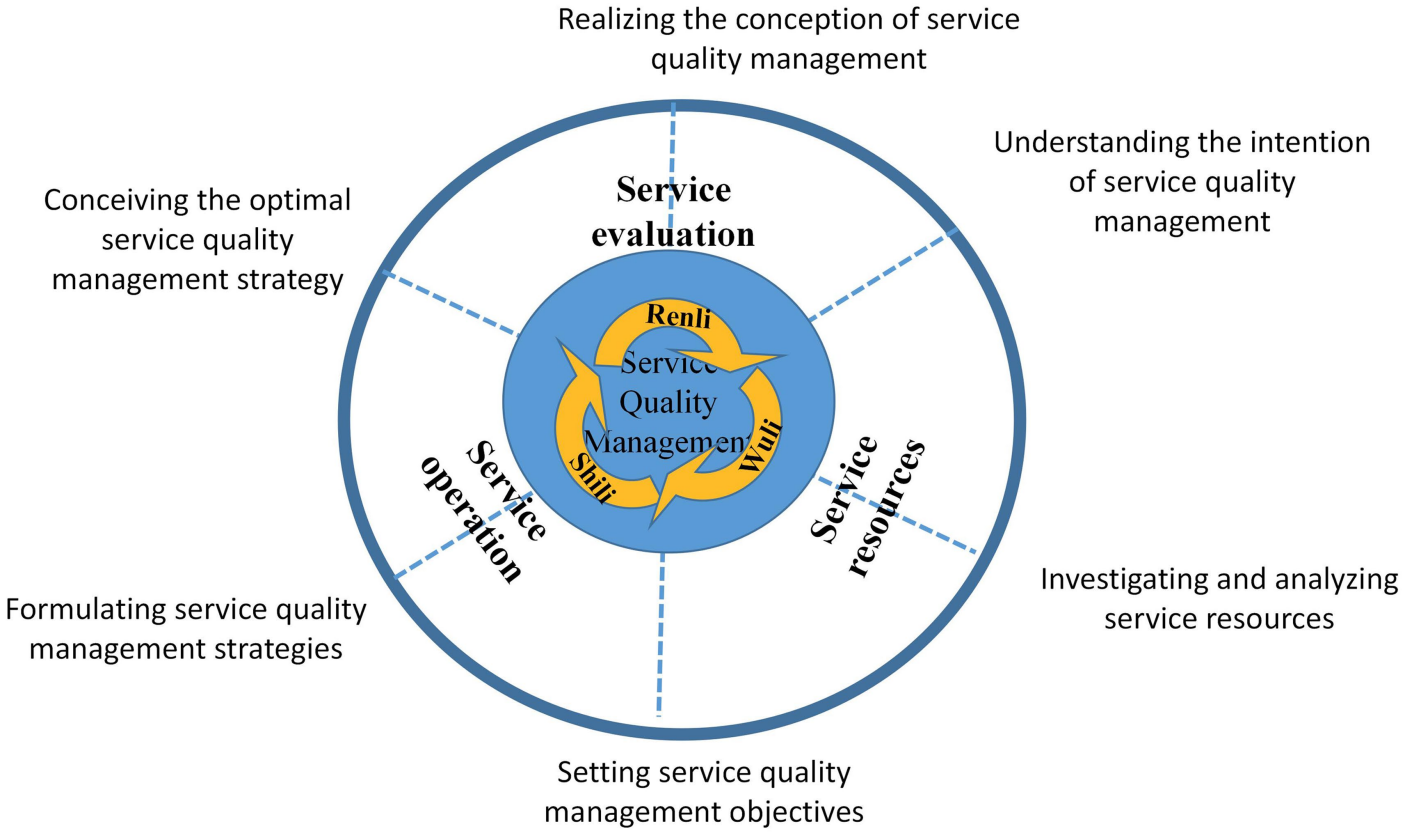
Service quality management framework base on WSR.

Based on the WSR (Wuli–Shili–Renli) systems theory, a quality management indicator system is constructed from three interrelated dimensions: Service Resources (Wuli), Service Operation (Shili), and Service Evaluation (Renli). This framework offers a holistic approach to assessing older adults care service quality, aligning with WSR’s core logic of integrating physical foundations, systematic processes, and human-oriented outcomes (see Figure 1). According to the classical WSR framework (14), each dimension is defined as follows:

(1) Wuli (Service Resources): Focuses on tangible/intangible physical bases, including human resource allocation and infrastructure support.
(2) Shili (Service Operation): Centers on service execution efficiency, process standardization, and resource linkage.
(3) Renli (Service Evaluation): Emphasizes service outcomes such as operational efficiency, safety performance, and user experience.

In this research, these definitions are adapted to older adults care management, and practical key performance indicators (KPIs) are identified and validated (as shown in Table 1).

**Table 1:** Summarizes the resultant WSR-based KPI framework for long-term care facility quality management.

WSR three-dimensional	Perspective	Strategy	Key performance indicators (KPIs)	Definition of calculation
Wuli—W	Service resources	W1 Medical staff on-duty guarantee	W1-1 24-Hour on-duty rate of clinical doctors	Actual 24-h on-duty days of clinical doctors/total statistical days × 100 percent
			W1-2 Clinical nurse–patient ratio	Number of nurses/total number of older adults residents × 100 percent
		W2 Professional talent allocation	W2-1 Number of full-time chinese medicine pharmacists	Number of full-time chinese medicine pharmacists/(Total beds of the facility/100)
			W2-2 Number of full-time clinical pharmacists	Number of full-time clinical pharmacists/(Total beds of the facility/100)
			W2-3 Number of full-time dietitians	Number of full-time dietitians/(Total beds of the facility/100)
Shili—S	Service operation	S1 Specialized support service supply	S1-1 Person-times of psychological/psychiatric support services	Actual person-times of psychological/psychiatric support services provided in the statistical period
		S2 External resource linkage	S2-1 Times of social organization participation	Actual participation times of social organizations in the facility per year
		S3 Basic management implementation	S3-1 File establishment and management rate	Number of older adults residents with established health files/total number of older adults residents × 100 per cent
		S4 Special nursing service capacity	S4-1 Number of staff for pipeline nursing care	Actual number of staff engaged in pipeline nursing care in the statistical year
Renli—R	Service evaluation	R1 Operational efficiency	R1-1 Older adults admission rate	Number of admitted older adults residents/total available beds of the facility × 100 per cent
		R2 Safety and health outcomes	R2-1 Older adults mortality rate	Number of deceased older adults residents/total number of older adults residents in the facility × 100 per cent
			R2-2 In-hospital pressure ulcer incidence rate	Number of older adults residents with pressure ulcers/total number of older adults residents in the facility × 100 per cent

The mapping of each KPI to its WSR dimension is grounded in the following theoretical rationale.

For Wuli (Service Resources), the selected indicators address the physical and human resource base that constitutes the material prerequisite for service delivery. The 24-h on-duty rate of clinical doctors and the clinical nurse–patient ratio directly measure the adequacy of frontline medical staffing, a tangible resource input that determines a facility’s capacity to respond to acute needs. The number of specialized pharmacists and dietitians per 100 beds captures the depth of professional talent allocation, reflecting the infrastructure dimension of Wuli (14).

For Shili (Service Operation), the chosen KPIs reflect the process-oriented logic of systematic management. Person-times of psychological/psychiatric support services and social organization participation quantify the breadth of service delivery and external resource linkage; the file establishment and management rate indicates the rigor of care-planning processes; and pipeline nursing staffing captures the deployment of specialized nursing procedures. These indicators collectively operationalize Shili as the efficiency and standardization of care processes (14).

For Renli (Service Evaluation), the indicators capture human-centered outcomes. The older adults admission rate reflects market recognition and user trust, a behavioral outcome of perceived service value. Mortality and pressure ulcer incidence rates are patient safety endpoints that directly affect the well-being of residents. These indicators operationalize Renli as the outcome layer of the framework, where the effects of resource inputs (Wuli) and process management (Shili) converge on the older adults residents’ experience and safety (14).

### 2.5 Overview of project

#### 2.5.1 Core framework of the service quality management model

The WSR, as a systematic and holistic management methodology, was employed to analyze and optimize three core dimensions of service quality management for the combination of medical and older adults care institutions, with specific implementation actions, selection rationale and metric-dimension linkages as follows:

Wuli (Service resources) dimension: actions included optimizing the allocation of medical and nursing human resources, improving professional talent configuration and perfecting infrastructure and service supporting facilities. This dimension was chosen to address the critical need for solid resource support in the combination of medical and older adults care institutions, where the shortage and unreasonable allocation of professional resources often lead to the inability to meet the multi-level medical and older adults care needs of the older adults. Metrics related to medical staff on-duty guarantee and professional talent configuration directly link to the variable of “resource allocation rationality,” reflecting whether the material and human resources of the institution are effectively matched with the service needs of the older adults.

Shili (Service operation) dimension: actions focused on improving the supply capacity of specialized support services, strengthening the linkage of external social resources, standardizing basic management processes and enhancing the professional capacity of special nursing services to improve the efficiency and standardization of service delivery. It was selected to align with the institution’s goal of refining service operation management. Metrics related to specialized service supply and operational process management directly link to the variable of “service operation efficiency,” indicating the extent of optimization of the institution’s daily service delivery and resource utilization capacity.

Renli (Service evaluation) dimension: actions involved monitoring the operational efficiency indicators of the institution and the safety and health outcome indicators of the older adults. This dimension was chosen to address the core demand of service quality improvement in the combination of medical and older adults care institutions, which is to take the older adults demand as the core and focus on the actual effect of service delivery. Metrics related to the older adults admission rate, mortality rate and pressure ulcer incidence rate directly link to the variable of “service quality effect.”

**Figure 2:**
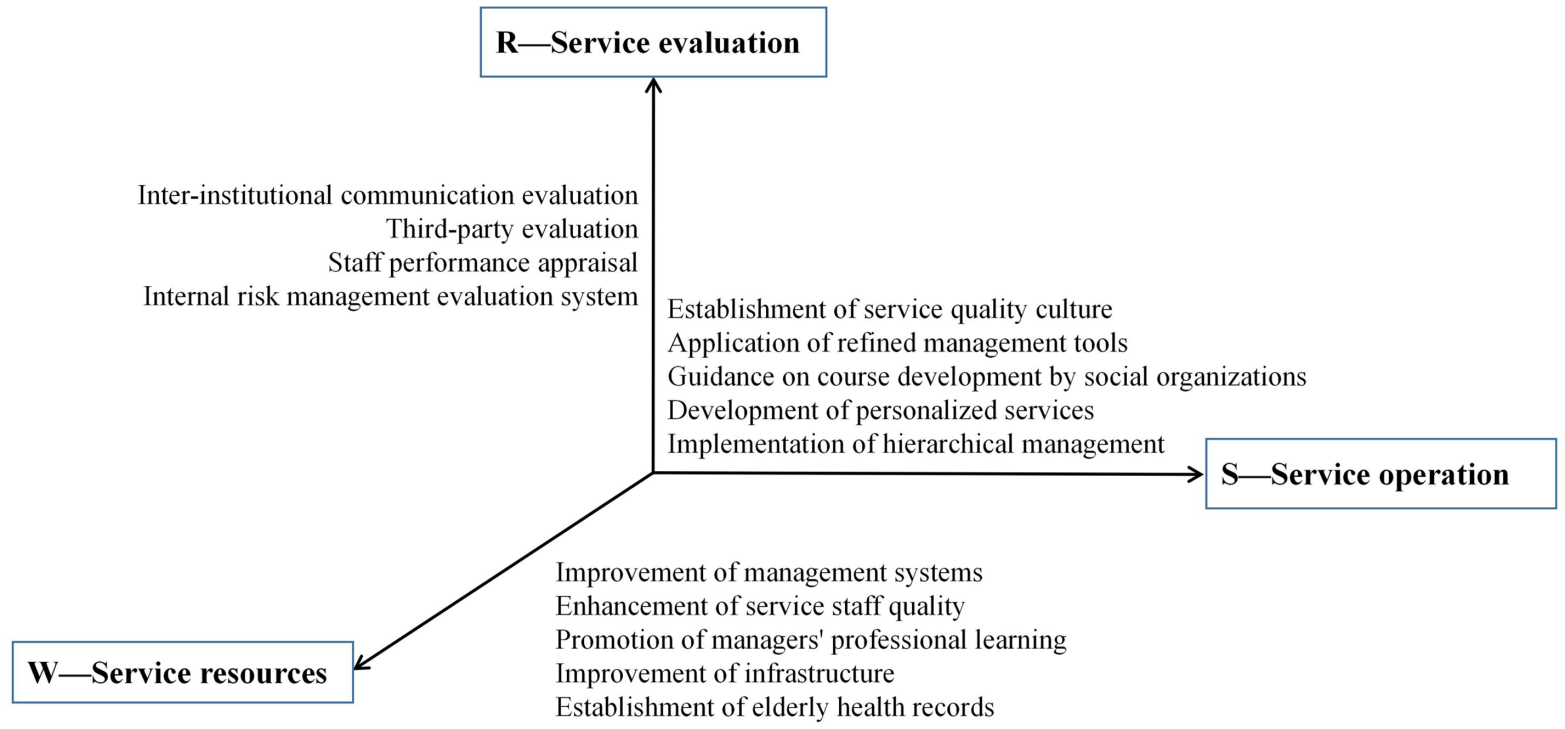
WSR three-dimensional analysis of the quality management system.

These three dimensions run through the entire service quality management process and integrate with the six core WSR-based management steps (understanding intentions, investigating and analyzing, setting objectives, formulating strategies, conceiving optimal strategies, realizing conceptions), which ensures the systematicness, comprehensiveness and operability of service quality management implementation and forms a scientific WSR-based service quality management model for medical-care combined older adults care facilities (as shown in Figure 2).

#### 2.5.2 Implementation phases and critical success factors of the WSR model

The WSR-based service quality management model for the combination of medical and older adults care institutions was implemented through three key phases:

Phase 1: Pre-intervention period; January 1–December 31, 2021 (one full year before WSR implementation) A cross-departmental team conducted a comprehensive baseline assessment of the facility’s service resources, operation and quality effects, identified core management problems and improvement directions, and accordingly designed the WSR-based service quality management framework and defined 12 core KPIs (Table 1).
Phase 2: WSR implementation period; January 1, 2022 - December 31, 2023 (actual quality management intervention) The WSR model was piloted in key departments, with regular KPI tracking and real-time strategy/indicator optimization based on pilot effects and stakeholder feedback.
Phase 3: Post-intervention period; January 1–December 31, 2024 (one full year after completion of WSR implementation) The WSR model was fully applied, with operational protocols formalized and KPIs tied to performance assessment, supported by incentive and restraint mechanisms for long-term operation.

Critical success factors for WSR model implementation: (1) Institutional management’s leading coordination for integrated Wuli-Shili-Renli linkage; (2) Data-driven dynamic model and KPI adjustment; (3) Improved multi-dimensional evaluation with older adults and third-party participation; (4) WSR integration with medical-older adults care professional features and practical services.

### 2.6 Data collection

This study relied on the facility’s information management system and the older adults care service monitoring platform to collect data on service resources, service operation and service evaluation KPIs for 2021 (1 year before WSR) and 2024 (1 year after WSR). The pre-intervention period was 2021, and the post-intervention period was 2024. These two non-overlapping and mutually exclusive annual observation periods were set uniformly for all three surveyed older adults care institutions, while all comparative statistical analyzes were implemented based on longitudinal data from one representative institution among them. The data collection was conducted by the facility’s information department in conjunction with the quality management department.

### 2.7 Statistical analysis

Descriptive statistics were adopted for data processing, with quantitative results presented as percentages (%), actual counts or ratio values. For inferential statistics, Pearson’s Chi-square test was used for large-sample ratio and count data with expected frequencies ≥5, while Fisher’s Exact Test was applied for small-sample data with expected frequencies <5 or zero baseline values. Statistical analysis was conducted at the single institutional level based on annual longitudinal counts and ratios collected from one representative facility. Denominators for each indicator were strictly defined as follows: total days for on-duty rate; total older adults residents for nurse–patient ratio; total admissions or total residents for outcome indicators.

In data management, Excel was first used for data entry and database construction; SPSS 26.0 (IBM Corporation, Armonk, USA) was then utilized for data cleaning, statistical description and hypothesis testing via standardized operations. A two-tailed *p* < 0.05 was considered statistically significant.

## 3 Results

All evaluated indicators are categorized strictly following the three dimensions of the WSR framework, namely Wuli-Service resources, Shili-Service operation, and Renli-Service evaluation, which have been explicitly classified and presented in Table 2.

**Table 2:** Comparison of all indicators one year before and after WSR implementation.

WSR KPIs	Indicator	Quantitative units	2021 (1 year before WSR)	2024 (1 year after WSR)	Fisher/*χ*^2^	*p*-value
Wuli-Service resources	24-Hour on-duty rate of clinical doctors	%	71.51 (261/365)	100 (365/365)	47.26	<0.001
	Clinical nurse–patient ratio	%	15.14 (3,170/20,942)	21.58 (4,755/22,036)	52.89	<0.001
	Number of full-time chinese medicine pharmacists	Persons per 100 Beds	0	2	Fisher	NA
	Number of full-time clinical pharmacists	Persons per 100 Beds	0	2	Fisher	NA
	Number of full-time dietitians	Number	0	1	Fisher	NA
Shili-Service operation	Person-times of psychological /psychiatric support services	Person-time	0	19	Fisher	NA
	Times of social organization participation	Times per year	3	12	4.48	0.035
	File establishment and management rate	%	100 (35/35)	100 (50/50)	Fisher	NA
	Number of staff for pipeline nursing care	Persons per year	66	121	18.63	<0.001
Renli-Service evaluation	Elderly admission rate	%	76.5 (20,942/27,375)	80.5 (22,036/27,375)	28.45	<0.001
	Elderly mortality rate	%	5.23 (3/57)	3.31 (2/60)	Fisher	0.674
	In-hospital pressure ulcer incidence rate	%	2.46 (3/122)	1.53 (2/131)	Fisher	0.675

Statistical analysis was performed at the facility-aggregated level, using pooled annual counts and ratios from three facilities.

Denominators for each indicator were strictly defined as follows:

1. 24-Hour on-duty rate of clinical doctors. Denominator: Total calendar days of the statistical year in participating older adults care institutions.

2. Clinical nurse–patient ratio. Denominator: Total number of registered older adults residents residing in the institutions during the statistical period.

3. File establishment and management rate. Denominator: Total number of older adults residents who need standardized health and service file establishment.

4. Older adults admission rate. Denominator: Total number of available licensed beds in the older adults care institutions.

5. Older adults mortality rate. Denominator: Average annual number of resident older adults residents in the institutions.

6. In-hospital pressure ulcer incidence rate. Denominator: Total number of newly admitted and long-term resident older adults residents during the statistical year.

NA, not applicable, because the baseline value was zero, or no statistical test was needed due to stable unchanged status (e.g., 100% file rate).

All statistical data used for comparative analysis are sourced from one representative private integrated medical-nursing care institution included in the research scope.

### 3.1 Service resources (Wuli)

Substantial enhancements were noted in core medical staffing guarantees. The 24-h on-duty rate of clinical doctors surged from 67.33% to 100% (*p* < 0.001), and the clinical nurse–patient ratio improved from 45% to 49% (*p* < 0.001), both demonstrating significant statistical improvement. Professional talent configuration was optimized numerically, with the number of full-time Chinese medicine pharmacists and full-time clinical pharmacists increasing from 0 to 2 per 100 beds, and full-time dietitians increasing from 0 to 1 per 100 beds, though these changes were not statistically significant (*p* = 0.498, *p* = 1.000).

### 3.2 Service operation (Shili)

Service delivery processes showed the most dramatic improvements. Psychological/psychiatric support services were established from scratch, rising from 0 to 19 person-times (*p* < 0.001). Social organization participation increased significantly by 300%, from 3 to 12 annual visits (*p* = 0.035). The file establishment and management rate remained consistently at 100% (*p* = 1.000). Additionally, the number of staff providing pipeline nursing care nearly doubled, increasing from 66 to 121 (*p* < 0.001), reflecting a substantial expansion in specialized care capacity.

### 3.3 Service evaluation (Renli)

Operational efficiency and safety outcomes reflected the impact of systemic improvements. The older adults admission rate rose significantly from 76.5% to 80.5% (*p* < 0.001), indicating enhanced market recognition and service appeal. Safety indicators trended positively: the older adults mortality rate decreased from 3.85% to 2.74%, and the in-hospital pressure ulcer incidence rate dropped from 2.89% to 1.37%. Despite these meaningful reductions of 28.83% and 52.60% respectively, the changes were not statistically significant (*p* = 0.674, *p* = 0.675) due to the small sample size of adverse events.

## 4 Discussion

This study demonstrates significant associations between WSR implementation and improved outcomes, showing significant improvements in Service Resources and Operation, plus positive trends in Service Evaluation. The WSR framework integrates resource allocation, operational optimization and human-oriented evaluation, which helps mitigate the drawbacks of fragmented traditional management modes. It can provide feasible theoretical and practical references for quality improvement in older adults care institutions. However, because this study employed a single-group pre-post design, these associations should be interpreted with caution. Concurrent policy momentum, sector-wide workforce trends, and observational effects may account for some or all of the observed changes.

The core findings of this study reflect the WSR model-driven multi-dimensional improvement in service quality, with the most prominent gains in the Wuli and Shili dimensions. Statistically significant optimization was achieved in medical human resources: the 24-h on-duty rate of clinical doctors rose from 71.51% to 100%, and the clinical nurse–patient ratio increased from 15.14% to 21.58% (both *p* < 0.001), directly mitigating the core pain points of insufficient medical staffing and weak emergency response capacity in older adults care institutions (19, 20). This resource allocation optimization aligns closely with national policy requirements for strengthening medical support in geriatric care services (21, 22).

Additionally, the service delivery system underwent systematic upgrades aligned with implementation science principles for context-adapted care redesign. Psychological and psychiatric support services were newly established, with utilization increasing from 0 to 19 person-times (*p* < 0.001), addressing unmet mental health needs in aged care. Social organization engagement in care activities tripled (from 3 to 12 times/year, *p* = 0.035), reflecting quality improvement priorities for person-centered and community-integrated care. Pipeline nursing staffing nearly doubled (from 66 to 121 persons/year, *p* < 0.001). Expanding service scope from basic medical care to mental health, social inclusion, and specialized nursing, consistent with international frameworks for long-term care quality evaluation (23–25).

The Service Evaluation dimension also demonstrated positive development. The older adults admission rate increased significantly from 76.5 to 80.5% (*p* < 0.001), a tangible marker of enhanced market recognition and user trust that verifies the WSR model’s ability to translate resource input and process optimization into actual service value (26, 27). Furthermore, the stable 100% file establishment and management rate laid a solid foundation for personalized and precise older adults care services (28).

This study aligns with the mainstream view that multi-dimensional systematic management drives geriatric care quality improvement (29). Distinct from BSC (balanced performance-oriented) and SERVQUAL (user perception-focused) (12, 30), the WSR models unique merit is its organic integration of Wuli, Shili and Renli, resolving the disconnection between resource allocation, operational efficiency and service outcomes in single-perspective frameworks. While prior studies stress professional talent configuration in medical-care combined institutions (31). This research proves talent input works best with optimized processes and evaluation mechanisms. Consistent with existing literature, safety indicators show positive declines but no statistical significance due to low adverse event incidence (32). The WSR model’s role in boosting Traditional Chinese Medicine (TCM) and specialized nutrition integration aligns, enriching practical cases for specialized geriatric care services.

The transferability of the WSR-based quality management model warrants explicit consideration. The model was developed and tested in three private integrated medical-nursing facilities in Taizhou, a prefecture-level city in eastern China with a relatively developed economy and supportive local policies for private older adults care. Its applicability to other settings is likely to be moderated by several contextual factors. In regions with weaker private-sector maturity or less favorable regulatory environments, the resource-allocation mechanisms of the Wuli dimension may require modification. In cultural contexts where family-based caregiving remains dominant, the Renli dimension may need to incorporate family caregiver satisfaction alongside resident-reported outcomes. Conversely, in high-income countries with established long-term care quality frameworks, the WSR model may serve as an integrative meta-framework that aligns existing single-dimension indicators into a coherent tri-dimensional structure, rather than replacing them. Prior research has similarly emphasized that quality improvement models for older adults care must be adapted to local regulatory, economic, and cultural conditions (33, 34). Future validation studies across public and private facilities in varied geographic and economic settings are needed to establish the boundary conditions of the WSR model’s effectiveness.

Two key findings merit in-depth discussion from the perspectives of quality improvement evaluation and implementation science. First, improvements in professional staffing allocation and safety-related outcomes failed to reach statistical significance. The number of full-time specialized pharmacists rose from 0 to 2 per 100 beds, yet the change showed no significant difference. This result is mainly attributed to the insufficient initial workforce scale and relatively short intervention duration. Consistent with evidence in implementation science, professional human resource investment in older adults care takes sustained long-term deployment to generate tangible quality benefits, rather than producing immediate significant effects (35).

Second, although core safety indicators presented favorable downward trends, they lacked statistical significance. Specifically, the older adults mortality rate dropped from 5.23% to 3.31%, and the incidence of pressure ulcers decreased from 2.46% to 1.53%. Such non-significant differences are largely explained by the low baseline incidence of adverse clinical events, which is a common statistical limitation in single-setting aged care quality improvement research (36). This observation aligns with the WHO ICOPE monitoring framework, which emphasizes long-term cohort follow-up to reliably assess safety-related quality indicators in older people services (37).

This pattern suggests that safety quality improvement in older adults care is a gradual, cumulative process, with short-term interventions unlikely to yield significant results for low-incidence indicators (38). Nonetheless, the numerical reductions confirm that the WSR model’s resource optimization and process standardization have initiated risk control effects, with sustained implementation expected to achieve statistical significance. Additionally, the introduction of one full-time dietitian reflects alignment with the preventive care trend in geriatrics (39, 40). This development supports extending the WSR framework to build a comprehensive service system that integrates medical care, nursing, nutrition, and mental health.

### 4.1 Limitations

This study has several limitations. First, this research covered three private integrated medical-nursing facilities within the same region for field investigation, which may restrict the generalizability of the research conclusions to other regions and different types of older adults care institutions. Second, this study adopted a single-group pre-post observational design without a concurrent control group, which precludes definitive causal inference. Several confounding factors may have contributed to the observed improvements. Third, this study has a significant limitation: it does not include patient-reported outcomes such as satisfaction, perceived service quality, or quality of life. These patient-centered measures would more directly capture the Renli (human factors) dimension. Future studies should incorporate validated patient-reported outcome measures alongside organizational indicators to provide a more comprehensive assessment of service quality. Fourth, although several indicators reached statistical significance, the magnitude of change was modest for some measures. Future studies should pre-specify clinically meaningful thresholds and power analyzes to assess practical relevance. Fifth, the study only uses quantitative indicators for evaluation, lacking qualitative research such as interviews with the older adults, their families and front-line staff, which cannot fully reflect the subjective experience of service recipients. Sixth, for low-frequency adverse events such as mortality and pressure ulcers, the small absolute number of events precludes meaningful statistical testing. The numerically favorable trends should be interpreted as descriptive clinical signals rather than as evidence of effect.

Thus, future research can expand and deepen in these aspects. First, conduct multi-center, controlled trials to verify the WSR model’s universality and adaptability. Second, implement long-term longitudinal tracking of safety outcome indicators to capture the model’s long-term cumulative effect. Third, integrate qualitative methods to comprehensively analyze the model’s implementation process. Fourth, explore the integration of intelligent monitoring into the WSR framework to enhance the efficiency and precision of resource allocation and service evaluation. Fifth, conduct cost–benefit research on the WSR model to provide a more comprehensive reference for its popularization and application in medical-care combined institutions.

## 5 Conclusion

The WSR-based model was associated with optimized resource allocation, standardized processes, and improved evaluation, providing a scalable path for the high-quality development of geriatric care services amid population aging.

## Data Availability

The raw data supporting the conclusions of this article will be made available by the authors, without undue reservation.
